# *N*,*N*′-Bis(2-chloro­benz­yl)-*N*′′-(dichloro­acet­yl)phospho­ric triamide

**DOI:** 10.1107/S1600536811000845

**Published:** 2011-01-12

**Authors:** Mehrdad Pourayoubi, Maryam Toghraee, Vladimir Divjakovic

**Affiliations:** a Department of Chemistry, Ferdowsi University of Mashhad, Mashhad 91779, Iran; b Department of Physics, Faculty of Sciences, University of Novi Sad, 21000, Serbia

## Abstract

In the title compound, C_16_H_16_Cl_4_N_3_O_2_P, the phosphoryl and carbonyl groups are *anti* to each other. The dihedral angle between the benzene rings is 33.59 (16)°. In the crystal, adjacent mol­ecules are linked *via* N—H⋯O=P and N—H⋯O=C hydrogen bonds, into an extended chain running parallel to the *a* axis.

## Related literature

For biologically active organo­phospho­rus compounds, see: Ekstrom *et al.* (2006 ▶). For the anti­cancer activity of compounds with a C(O)NHP(O) skeleton, see: Gholivand *et al.* (2011 ▶). For related structures, see: Sabbaghi *et al.* (2010**a* ▶,b* ▶). 
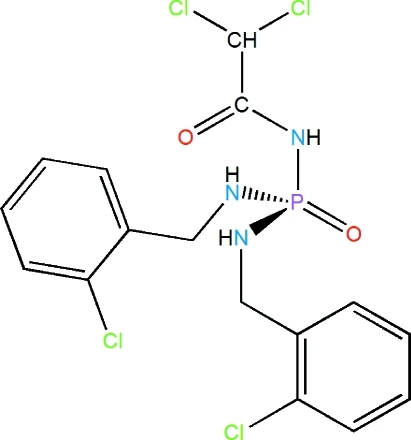


## Experimental

### 

#### Crystal data

C_16_H_16_Cl_4_N_3_O_2_P
*M* *_r_* = 455.09
Triclinic, 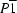

*a* = 9.901 (1) Å
*b* = 10.179 (1) Å
*c* = 12.013 (2) Å
α = 90.403 (5)°
β = 112.851 (6)°
γ = 114.084 (6)°
*V* = 998.7 (2) Å^3^
*Z* = 2
Mo *K*α radiation
μ = 0.69 mm^−1^
*T* = 295 K
0.22 × 0.12 × 0.11 mm

#### Data collection

Oxford Diffraction Xcalibur Sapphire3 Gemini diffractometer
Absorption correction: multi-scan (*CrysAlis PRO*; Oxford Diffraction, 2009 ▶) *T*_min_ = 0.978, *T*_max_ = 1.000
6193 measured reflections
3510 independent reflections
2786 reflections with *I* > 2σ(*I*)
*R*_int_ = 0.018

#### Refinement

*R*[*F* ^2^ > 2σ(*F* ^2^)] = 0.061
*wR*(*F* ^2^) = 0.156
*S* = 1.02
3510 reflections
235 parameters
H-atom parameters constrained
Δρ_max_ = 0.96 e Å^−3^
Δρ_min_ = −0.65 e Å^−3^

### 

Data collection: *CrysAlis PRO* (Oxford Diffraction, 2009 ▶); cell refinement: *CrysAlis PRO*; data reduction: *CrysAlis PRO*; program(s) used to solve structure: *SIR92* (Altomare *et al.*, 1993 ▶); program(s) used to refine structure: *SHELXL97* (Sheldrick, 2008 ▶); molecular graphics: *Mercury* (Macrae *et al.*, 2008 ▶); software used to prepare material for publication: *SHELXL97* and *PLATON* (Spek, 2009 ▶).

## Supplementary Material

Crystal structure: contains datablocks I, global. DOI: 10.1107/S1600536811000845/fi2102sup1.cif

Structure factors: contains datablocks I. DOI: 10.1107/S1600536811000845/fi2102Isup2.hkl

Additional supplementary materials:  crystallographic information; 3D view; checkCIF report

## Figures and Tables

**Table 1:** Hydrogen-bond geometry (Å, °)

*D*—H⋯*A*	*D*—H	H⋯*A*	*D*⋯*A*	*D*—H⋯*A*
N2—H2⋯O1^i^	0.86	1.93	2.756 (4)	162
N3—H3⋯O2^ii^	0.86	2.24	3.024 (4)	151

Symmetry codes: (i) 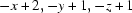; (ii) 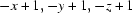.

**Fig. 1.**
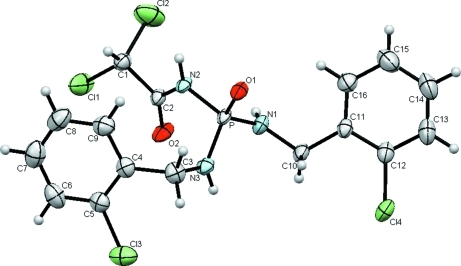
An ORTEP style plot of title compound. Ellipsoids are given at 30% probability level.

## supplementary crystallographic information

### Comment

Organophosphorus compounds are well-known as the biologically active substances (Ekstrom *et al.*, 2006). Among them the anticancer activity of compounds having a C(O)NHP(O) skeleton has been studied (Gholivand *et al.*, 2011). In the previous works, some phosphoric triamides such as P(O)[NHC(O)C_6_H_4_(4-NO_2_)][NHC_6_H_11_]_2_ (Sabbaghi *et al.*, 2010*a*) and P(O)[NHC(O)C_6_H_4_(4-NO_2_)][N(CH_3_)(C_6_H_11_)]_2_ (Sabbaghi *et al.*, 2010*b*) have been structurally investigated. We report here on the synthesis and crystal structure of P(O)[NHC(O)CHCl_2_][NHCH_2_(2-Cl—C_6_H_4_)]_2_. Single crystals of title compound were obtained from a solution of CH_3_OH and CH_3_CN after a slow evaporation at room temperature. The phosphoryl and carbonyl groups are *anti* to each other and the phosphorus atom is in a slightly distorted tetrahedral environment (Fig. 1). The bond angles are in the range of 103.08 (16)°-117.84 (17)° around the P atom. The P—N1 and P—N3 (1.616 (3) Å and 1.619 (3) Å) bond lengths are shorter than the P—N2 bond (1.682 (3) Å). The environment of nitrogen atoms is essentially planar. The P═O bond length of 1.471 (3) Å is standard for phosphoramidate compounds.

In the crystal structure, adjacent molecules are linked *via* N–H···O═P and N–H···O═C hydrogen bonds, into an extended chain parallel to the *a* axis.

### Experimental

The reaction of phosphorus pentachloride (16.91 mmol) and CHCl_2_C(O)NH_2_ (16.91 mmol) in dry CCl_4_ at 358 K (3 h) and then the treatment of formic acid (16.91 mmol) at ice bath temperature leads to CHCl_2_C(O)NHP(O)Cl_2_.

To a solution of CHCl_2_C(O)NHP(O)Cl_2_ (1.04 mmol) in dry CHCl_3_, a solution of 2-chlorobenzylamine (4.16 mmol) in dry CHCl_3_ was added dropwise and stirred at 273 K. After 4 h, the solvent was evaporated at room temperature. The solid was washed with H_2_O. The product was obtained after recrystallization from a methanol/acetonitrile mixture (4:1) after a slow evaporation at room temperature. IR (KBr, cm^-1^): 3392 (NH), 3080 (NH), 2881, 1704 (C═O), 1465, 1203 (P═O), 1072, 887.

### Refinement

All H atoms were placed at calculated positions and were refined riding on the respective carrier atoms.

### Crystal data

**Table:**

C_16_H_16_Cl_4_N_3_O_2_P	*Z* = 2
*M**_r_* = 455.09	*F*(000) = 464
Triclinic, *P*1	*D*_x_ = 1.513 Mg m^−^^3^
Hall symbol: -P 1	Mo *K*α radiation, λ = 0.71073 Å
*a* = 9.901 (1) Å	Cell parameters from 2802 reflections
*b* = 10.179 (1) Å	θ = 3.5–29.0°
*c* = 12.013 (2) Å	µ = 0.69 mm^−^^1^
α = 90.403 (5)°	*T* = 295 K
β = 112.851 (6)°	Prism, colourless
γ = 114.084 (6)°	0.22 × 0.12 × 0.11 mm
*V* = 998.7 (2) Å^3^	

### Data collection

**Table:**

Oxford Diffraction Xcalibur Sapphire3 Gemini diffractometer	3510 independent reflections
Radiation source: Enhance (Mo) X-ray Source	2786 reflections with *I* > 2σ(*I*)
graphite	*R*_int_ = 0.018
Detector resolution: 16.3280 pixels mm^-1^	θ_max_ = 25.0°, θ_min_ = 3.5°
ω scans	*h* = −11→9
Absorption correction: multi-scan (*CrysAlis PRO*; Oxford Diffraction, 2009)	*k* = −11→12
*T*_min_ = 0.978, *T*_max_ = 1.000	*l* = −14→13
6193 measured reflections	

### Refinement

**Table:**

Refinement on *F*^2^	Primary atom site location: structure-invariant direct methods
Least-squares matrix: full	Secondary atom site location: difference Fourier map
*R*[*F*^2^ > 2σ(*F*^2^)] = 0.061	Hydrogen site location: inferred from neighbouring sites
*wR*(*F*^2^) = 0.156	H-atom parameters constrained
*S* = 1.02	*w* = 1/[σ^2^(*F*_o_^2^) + (0.0618*P*)^2^ + 1.5174*P*] where *P* = (*F*_o_^2^ + 2*F*_c_^2^)/3
3510 reflections	(Δ/σ)_max_ < 0.001
235 parameters	Δρ_max_ = 0.96 e Å^−^^3^
0 restraints	Δρ_min_ = −0.65 e Å^−^^3^

### Special details

**Table:**

Geometry. All e.s.d.'s (except the e.s.d. in the dihedral angle between two l.s. planes) are estimated using the full covariance matrix. The cell e.s.d.'s are taken into account individually in the estimation of e.s.d.'s in distances, angles and torsion angles; correlations between e.s.d.'s in cell parameters are only used when they are defined by crystal symmetry. An approximate (isotropic) treatment of cell e.s.d.'s is used for estimating e.s.d.'s involving l.s. planes.
Refinement. Refinement of *F*^2^ against ALL reflections. The weighted *R*-factor *wR* and goodness of fit *S* are based on *F*^2^, conventional *R*-factors *R* are based on *F*, with *F* set to zero for negative *F*^2^. The threshold expression of *F*^2^ > σ(*F*^2^) is used only for calculating *R*-factors(gt) *etc*. and is not relevant to the choice of reflections for refinement. *R*-factors based on *F*^2^ are statistically about twice as large as those based on *F*, and *R*- factors based on ALL data will be even larger.

### Fractional atomic coordinates and isotropic or equivalent isotropic displacement parameters (Å^2^)

**Table:**

	*x*	*y*	*z*	*U*_iso_*/*U*_eq_	
P	0.84901 (11)	0.52095 (11)	0.59850 (8)	0.0398 (3)	
Cl1	0.4017 (2)	0.3402 (3)	0.12756 (12)	0.1300 (8)	
Cl2	0.5672 (2)	0.16627 (16)	0.23126 (18)	0.1108 (6)	
Cl3	0.65788 (18)	0.91760 (16)	0.50769 (15)	0.0893 (5)	
Cl4	0.77087 (18)	0.44836 (16)	1.03178 (11)	0.0804 (4)	
O1	1.0238 (3)	0.5568 (3)	0.6484 (2)	0.0543 (7)	
O2	0.5072 (3)	0.4115 (4)	0.3908 (3)	0.0670 (9)	
N1	0.7379 (4)	0.4006 (4)	0.6531 (3)	0.0496 (8)	
H1	0.6627	0.3184	0.6048	0.059*	
N2	0.7656 (3)	0.4413 (3)	0.4495 (3)	0.0427 (7)	
H2	0.8279	0.4239	0.4240	0.051*	
N3	0.8251 (4)	0.6669 (3)	0.6149 (3)	0.0481 (8)	
H3	0.7538	0.6631	0.6405	0.058*	
C1	0.5775 (5)	0.3419 (5)	0.2368 (4)	0.0512 (10)	
H1A	0.6696	0.4058	0.2197	0.061*	
C2	0.6105 (4)	0.4020 (4)	0.3661 (3)	0.0423 (8)	
C3	0.9204 (5)	0.8049 (5)	0.5877 (4)	0.0611 (11)	
H3A	1.0292	0.8142	0.6081	0.073*	
H3B	0.9319	0.8859	0.6395	0.073*	
C4	0.8437 (5)	0.8158 (4)	0.4545 (4)	0.0524 (10)	
C5	0.7214 (5)	0.8603 (4)	0.4084 (4)	0.0560 (10)	
C6	0.6453 (6)	0.8610 (5)	0.2847 (5)	0.0741 (14)	
H6	0.5635	0.8919	0.2570	0.089*	
C7	0.6907 (8)	0.8161 (6)	0.2034 (5)	0.0846 (16)	
H7	0.6393	0.8155	0.1199	0.102*	
C8	0.8110 (8)	0.7727 (6)	0.2449 (5)	0.0837 (16)	
H8	0.8424	0.7428	0.1897	0.100*	
C9	0.8878 (6)	0.7724 (5)	0.3692 (5)	0.0678 (12)	
H9	0.9705	0.7425	0.3960	0.081*	
C10	0.7581 (5)	0.4233 (5)	0.7789 (4)	0.0513 (10)	
H10A	0.6585	0.4211	0.7776	0.062*	
H10B	0.8462	0.5203	0.8215	0.062*	
C11	0.7950 (4)	0.3128 (4)	0.8509 (3)	0.0458 (9)	
C12	0.8030 (5)	0.3159 (5)	0.9693 (4)	0.0571 (11)	
C13	0.8391 (6)	0.2189 (6)	1.0401 (5)	0.0745 (14)	
H13	0.8461	0.2247	1.1196	0.089*	
C14	0.8644 (7)	0.1145 (7)	0.9923 (6)	0.0904 (17)	
H14	0.8875	0.0476	1.0391	0.108*	
C15	0.8562 (7)	0.1062 (6)	0.8740 (6)	0.0893 (17)	
H15	0.8733	0.0343	0.8414	0.107*	
C16	0.8225 (6)	0.2060 (5)	0.8060 (5)	0.0657 (12)	
H16	0.8182	0.2013	0.7273	0.079*	

### Atomic displacement parameters (Å^2^)

**Table:**

	*U*^11^	*U*^22^	*U*^33^	*U*^12^	*U*^13^	*U*^23^
P	0.0347 (5)	0.0643 (6)	0.0311 (5)	0.0268 (5)	0.0192 (4)	0.0135 (4)
Cl1	0.1450 (15)	0.226 (2)	0.0426 (7)	0.1471 (16)	−0.0041 (8)	−0.0079 (9)
Cl2	0.1193 (13)	0.0728 (9)	0.1247 (14)	0.0485 (9)	0.0318 (11)	−0.0113 (9)
Cl3	0.0902 (10)	0.0832 (9)	0.1143 (12)	0.0446 (8)	0.0567 (9)	0.0103 (8)
Cl4	0.1089 (10)	0.1091 (10)	0.0488 (7)	0.0593 (9)	0.0469 (7)	0.0242 (6)
O1	0.0390 (14)	0.097 (2)	0.0371 (14)	0.0384 (15)	0.0170 (12)	0.0138 (14)
O2	0.0404 (15)	0.119 (3)	0.0490 (17)	0.0405 (17)	0.0219 (13)	0.0053 (16)
N1	0.0538 (19)	0.060 (2)	0.0360 (17)	0.0223 (16)	0.0241 (15)	0.0118 (14)
N2	0.0367 (16)	0.069 (2)	0.0332 (16)	0.0281 (15)	0.0206 (13)	0.0093 (14)
N3	0.0443 (17)	0.062 (2)	0.0473 (19)	0.0251 (16)	0.0272 (15)	0.0121 (15)
C1	0.050 (2)	0.064 (2)	0.041 (2)	0.029 (2)	0.0177 (18)	0.0055 (18)
C2	0.0390 (19)	0.060 (2)	0.0346 (19)	0.0248 (18)	0.0187 (16)	0.0131 (16)
C3	0.050 (2)	0.058 (3)	0.061 (3)	0.018 (2)	0.017 (2)	0.008 (2)
C4	0.048 (2)	0.045 (2)	0.060 (3)	0.0138 (18)	0.025 (2)	0.0131 (18)
C5	0.052 (2)	0.047 (2)	0.065 (3)	0.0174 (19)	0.027 (2)	0.012 (2)
C6	0.062 (3)	0.059 (3)	0.084 (4)	0.024 (2)	0.018 (3)	0.022 (3)
C7	0.089 (4)	0.073 (3)	0.067 (3)	0.019 (3)	0.029 (3)	0.016 (3)
C8	0.103 (4)	0.075 (3)	0.078 (4)	0.024 (3)	0.058 (3)	0.013 (3)
C9	0.069 (3)	0.062 (3)	0.086 (4)	0.029 (2)	0.046 (3)	0.021 (2)
C10	0.058 (2)	0.068 (3)	0.041 (2)	0.031 (2)	0.0303 (19)	0.0182 (19)
C11	0.039 (2)	0.059 (2)	0.041 (2)	0.0201 (18)	0.0203 (17)	0.0160 (17)
C12	0.050 (2)	0.075 (3)	0.047 (2)	0.027 (2)	0.0222 (19)	0.021 (2)
C13	0.070 (3)	0.092 (4)	0.059 (3)	0.034 (3)	0.027 (2)	0.038 (3)
C14	0.095 (4)	0.097 (4)	0.090 (4)	0.056 (4)	0.036 (3)	0.053 (3)
C15	0.097 (4)	0.088 (4)	0.112 (5)	0.059 (3)	0.053 (4)	0.046 (3)
C16	0.069 (3)	0.077 (3)	0.066 (3)	0.038 (3)	0.038 (2)	0.022 (2)

### Geometric parameters (Å, °)

**Table:**

P—O1	1.471 (3)	C5—C6	1.382 (7)
P—N1	1.616 (3)	C6—C7	1.367 (8)
P—N3	1.619 (3)	C6—H6	0.9300
P—N2	1.682 (3)	C7—C8	1.354 (8)
Cl1—C1	1.718 (4)	C7—H7	0.9300
Cl2—C1	1.748 (4)	C8—C9	1.388 (7)
Cl3—C5	1.741 (5)	C8—H8	0.9300
Cl4—C12	1.733 (5)	C9—H9	0.9300
O2—C2	1.208 (4)	C10—C11	1.500 (5)
N1—C10	1.450 (5)	C10—H10A	0.9700
N1—H1	0.8600	C10—H10B	0.9700
N2—C2	1.349 (4)	C11—C16	1.376 (6)
N2—H2	0.8600	C11—C12	1.394 (5)
N3—C3	1.461 (5)	C12—C13	1.375 (6)
N3—H3	0.8600	C13—C14	1.358 (8)
C1—C2	1.525 (5)	C13—H13	0.9300
C1—H1A	0.9800	C14—C15	1.392 (8)
C3—C4	1.509 (6)	C14—H14	0.9300
C3—H3A	0.9700	C15—C16	1.373 (7)
C3—H3B	0.9700	C15—H15	0.9300
C4—C5	1.381 (6)	C16—H16	0.9300
C4—C9	1.392 (6)		
			
O1—P—N1	117.84 (17)	C7—C6—C5	119.7 (5)
O1—P—N3	110.86 (18)	C7—C6—H6	120.1
N1—P—N3	106.48 (17)	C5—C6—H6	120.1
O1—P—N2	106.38 (15)	C8—C7—C6	119.7 (5)
N1—P—N2	103.08 (16)	C8—C7—H7	120.2
N3—P—N2	112.03 (16)	C6—C7—H7	120.2
C10—N1—P	123.7 (3)	C7—C8—C9	120.7 (5)
C10—N1—H1	118.2	C7—C8—H8	119.7
P—N1—H1	118.2	C9—C8—H8	119.7
C2—N2—P	126.3 (2)	C4—C9—C8	121.3 (5)
C2—N2—H2	116.9	C4—C9—H9	119.3
P—N2—H2	116.9	C8—C9—H9	119.3
C3—N3—P	122.2 (3)	N1—C10—C11	114.6 (3)
C3—N3—H3	118.9	N1—C10—H10A	108.6
P—N3—H3	118.9	C11—C10—H10A	108.6
C2—C1—Cl1	111.5 (3)	N1—C10—H10B	108.6
C2—C1—Cl2	109.2 (3)	C11—C10—H10B	108.6
Cl1—C1—Cl2	111.2 (2)	H10A—C10—H10B	107.6
C2—C1—H1A	108.3	C16—C11—C12	117.0 (4)
Cl1—C1—H1A	108.3	C16—C11—C10	123.2 (4)
Cl2—C1—H1A	108.3	C12—C11—C10	119.7 (4)
O2—C2—N2	123.9 (3)	C13—C12—C11	122.2 (5)
O2—C2—C1	123.1 (3)	C13—C12—Cl4	118.4 (4)
N2—C2—C1	113.0 (3)	C11—C12—Cl4	119.4 (3)
N3—C3—C4	113.0 (3)	C12—C13—C14	119.0 (5)
N3—C3—H3A	109.0	C12—C13—H13	120.5
C4—C3—H3A	109.0	C14—C13—H13	120.5
N3—C3—H3B	109.0	C15—C14—C13	120.8 (5)
C4—C3—H3B	109.0	C15—C14—H14	119.6
H3A—C3—H3B	107.8	C13—C14—H14	119.6
C5—C4—C9	116.2 (4)	C14—C15—C16	118.9 (5)
C5—C4—C3	123.2 (4)	C14—C15—H15	120.6
C9—C4—C3	120.5 (4)	C16—C15—H15	120.6
C4—C5—C6	122.5 (4)	C11—C16—C15	122.0 (5)
C4—C5—Cl3	119.7 (4)	C11—C16—H16	119.0
C6—C5—Cl3	117.8 (4)	C15—C16—H16	119.0
			
O1—P—N1—C10	66.5 (4)	C4—C5—C6—C7	0.1 (7)
N3—P—N1—C10	−58.7 (3)	Cl3—C5—C6—C7	−179.6 (4)
N2—P—N1—C10	−176.8 (3)	C5—C6—C7—C8	−0.6 (8)
O1—P—N2—C2	−174.1 (3)	C6—C7—C8—C9	0.4 (8)
N1—P—N2—C2	61.3 (4)	C5—C4—C9—C8	−0.7 (6)
N3—P—N2—C2	−52.8 (4)	C3—C4—C9—C8	175.8 (4)
O1—P—N3—C3	44.0 (3)	C7—C8—C9—C4	0.3 (8)
N1—P—N3—C3	173.4 (3)	P—N1—C10—C11	−121.2 (3)
N2—P—N3—C3	−74.6 (3)	N1—C10—C11—C16	5.6 (6)
P—N2—C2—O2	−4.7 (6)	N1—C10—C11—C12	−174.5 (4)
P—N2—C2—C1	176.0 (3)	C16—C11—C12—C13	1.0 (6)
Cl1—C1—C2—O2	17.2 (5)	C10—C11—C12—C13	−178.9 (4)
Cl2—C1—C2—O2	−106.1 (4)	C16—C11—C12—Cl4	179.6 (3)
Cl1—C1—C2—N2	−163.5 (3)	C10—C11—C12—Cl4	−0.3 (5)
Cl2—C1—C2—N2	73.2 (4)	C11—C12—C13—C14	−1.4 (7)
P—N3—C3—C4	87.6 (4)	Cl4—C12—C13—C14	179.9 (4)
N3—C3—C4—C5	83.3 (5)	C12—C13—C14—C15	0.8 (9)
N3—C3—C4—C9	−93.0 (5)	C13—C14—C15—C16	0.2 (9)
C9—C4—C5—C6	0.5 (6)	C12—C11—C16—C15	0.1 (7)
C3—C4—C5—C6	−175.9 (4)	C10—C11—C16—C15	180.0 (5)
C9—C4—C5—Cl3	−179.7 (3)	C14—C15—C16—C11	−0.7 (8)
C3—C4—C5—Cl3	3.9 (5)		

### Hydrogen-bond geometry (Å, °)

**Table:**

*D*—H···*A*	*D*—H	H···*A*	*D*···*A*	*D*—H···*A*
N2—H2···O1^i^	0.86	1.93	2.756 (4)	162
N3—H3···O2^ii^	0.86	2.24	3.024 (4)	151

Symmetry codes: (i) −*x*+2, −*y*+1, −*z*+1; (ii) −*x*+1, −*y*+1, −*z*+1.
